# The emerging spectrum of fetal acetylcholine receptor antibody-related disorders (FARAD)

**DOI:** 10.1093/brain/awad153

**Published:** 2023-05-15

**Authors:** Nicholas M Allen, Mark O’Rahelly, Bruno Eymard, Mondher Chouchane, Andreas Hahn, Gerry Kearns, Dae-Seong Kim, Shin Yun Byun, Cam-Tu Emilie Nguyen, Ulrike Schara-Schmidt, Heike Kölbel, Adela Della Marina, Christiane Schneider-Gold, Kathryn Roefke, Andrea Thieme, Peter Van den Bergh, Gloria Avalos, Rodrigo Álvarez-Velasco, Daniel Natera-de Benito, Man Hin Mark Cheng, Wing Ki Chan, Hoi Shan Wan, Mary Ann Thomas, Lauren Borch, Julie Lauzon, Cornelia Kornblum, Jens Reimann, Andreas Mueller, Thierry Kuntzer, Fiona Norwood, Sithara Ramdas, Leslie W Jacobson, Xiaobo Jie, Miguel A Fernandez-Garcia, Elizabeth Wraige, Ming Lim, Jean Pierre Lin, Kristl G Claeys, Selma Aktas, Maryam Oskoui, Yael Hacohen, Ameneh Masud, M Isabel Leite, Jacqueline Palace, Darryl De Vivo, Angela Vincent, Heinz Jungbluth

**Affiliations:** Department of Paediatrics, School of Medicine, University of Galway, Galway H91 V4AY, Ireland; Department of Paediatrics, School of Medicine, University of Galway, Galway H91 V4AY, Ireland; Centre de référence des maladies neuromusculaires Nord/Est/Ile-de-France, Unité Pathologie Neuromusculaire, Bâtiment Babinski, G.H. Pitie-Salpetriere, 75013 Paris, France; Department of Pediatrics, Centre Hospitalier Universitaire de Dijon, Dijon, France; Department of Child Neurology, University Hospital Giessen, 35392 Giessen, Germany; Department of Maxillofacial Surgery, St. James Hospital, Dublin D08 NHY1, Ireland; Department of Neurology, Pusan National University, School of Medicine, Pusan 50612, South Korea; Department of Pediatrics, Pusan National University, School of Medicine, Pusan 50612, South Korea; Pediatric Neurology, CHU Sainte-Justine and Département de neurosciences, Université de Montréal, QC, H3T 1C5, Canada; Department of Pediatric Neurology, Centre for Translational Neuro- and Behavioral Sciences, University Duisburg, Essen, DE-45147 Essen, Germany; Department of Pediatric Neurology, Centre for Translational Neuro- and Behavioral Sciences, University Duisburg, Essen, DE-45147 Essen, Germany; Department of Pediatric Neurology, Centre for Translational Neuro- and Behavioral Sciences, University Duisburg, Essen, DE-45147 Essen, Germany; Department of Neurology, St Josef Hospital, Ruhr-University Bochum, 44791 Bochum, Germany; Klinik für Kinder- und Jugendmedizin, 99089 Erfurt, Germany; Department of Neurology, Clinical Neurophysiology and Neurorehabilitation, St. Georg Klinikum, 99817 Eisenach, Germany; Neuromuscular Reference Centre UCL St-Luc, University Hospital Saint-Luc, 1200 Brussels, Belgium; Department of Medicine, University of Galway, Galway H91 V4AY, Ireland; Unitat Patologia Neuromuscular, Servei Neurologia Hospital Santa Creu i Sant Pau, 08025 Barcelona, Spain; Neuromuscular Unit, Hospital Sant Joan de Déu, 08950 Barcelona, Spain; Department of Paediatrics and Adolescent Medicine, Princess Margaret Hospital, Hong Kong; Department of Paediatrics and Adolescent Medicine, Princess Margaret Hospital, Hong Kong; Department of Paediatrics and Adolescent Medicine, Princess Margaret Hospital, Hong Kong; Department of Medical Genetics and Pediatrics, Cumming School of Medicine, University of Calgary, Alberta Children’s Hospital, Calgary, AB T3B 6A8, Canada; Department of Medical Genetics and Pediatrics, Cumming School of Medicine, University of Calgary, Alberta Children’s Hospital, Calgary, AB T3B 6A8, Canada; Department of Medical Genetics and Pediatrics, Cumming School of Medicine, University of Calgary, Alberta Children’s Hospital, Calgary, AB T3B 6A8, Canada; Department of Neurology, University Hospital Bonn, 53127 Bonn, Germany; Center for Rare Diseases, University Hospital Bonn, 53127 Bonn, Germany; Department of Neurology, University Hospital Bonn, 53127 Bonn, Germany; Department of Neonatology and Pediatric Intensive Care, University Hospital Bonn, 53127, Bonn, Germany; Nerve-Muscle Unit, Department of Clinical Neurosciences, CHUV, University of Lausanne, 1011 Lausanne, Switzerland; Department of Neurology, King’s College Hospital, London SE5 9RS, UK; MDUK Neuromuscular Centre, Department of Paediatrics, University of Oxford, Oxford OX3 9DU, UK; Nuffield Department of Clinical Neurosciences, Oxford University, Oxford OX3 9DU, UK; Nuffield Department of Clinical Neurosciences, Oxford University, Oxford OX3 9DU, UK; Department of Children’s Neurosciences, Evelina London Children's Hospital, Guy’s & St. Thomas’ Hospital NHS Foundation Trust, London SE1 7EH, UK; Department of Children’s Neurosciences, Evelina London Children's Hospital, Guy’s & St. Thomas’ Hospital NHS Foundation Trust, London SE1 7EH, UK; Department of Children’s Neurosciences, Evelina London Children's Hospital, Guy’s & St. Thomas’ Hospital NHS Foundation Trust, London SE1 7EH, UK; Department of Women and Children’s Health, School of Life Course Sciences (SoLCS), King’s College London, London SE1 9NH, UK; Department of Children’s Neurosciences, Evelina London Children's Hospital, Guy’s & St. Thomas’ Hospital NHS Foundation Trust, London SE1 7EH, UK; Department of Neurology, University Hospitals Leuven, 3000 Leuven, Belgium; Laboratory for Muscle Diseases and Neuropathies, Department of Neurosciences, KU Leuven, and Leuven Brain Institute (LBI), 3000 Leuven, Belgium; Faculty of Medicine, Department of Pediatrics, Division of Neonatology, Acıbadem University, 34752 Istanbul, Turkey; Department of Pediatrics, McGill University, Montreal, QC H4A 3J1, Canada; Department of Neurology and Neurosurgery, McGill University, Montreal, QC H4A 3J1, Canada; Centre for Outcomes Research and Evaluation, Research Institute McGill University Health Centre, Montreal, QC H3H 2R9, Canada; Queen Square MS Centre, UCL Queen Square Institute of Neurology, Faculty of Brain Sciences, University College London, London WC1N 3BG, UK; Department of Neurology, Great Ormond Street Hospital for Children, London WC1N 3JH, UK; Department of Neurology, Columbia University Irving Medical Center, New York, NY 10032-3791, USA; Department of Pediatrics, Columbia University Irving Medical Center, New York, NY 10032-3791, USA; Nuffield Department of Clinical Neurosciences, Oxford University, Oxford OX3 9DU, UK; Nuffield Department of Clinical Neurosciences, Oxford University, Oxford OX3 9DU, UK; Department of Neurology, Columbia University Irving Medical Center, New York, NY 10032-3791, USA; Department of Pediatrics, Columbia University Irving Medical Center, New York, NY 10032-3791, USA; Nuffield Department of Clinical Neurosciences, Oxford University, Oxford OX3 9DU, UK; Department of Children’s Neurosciences, Evelina London Children's Hospital, Guy’s & St. Thomas’ Hospital NHS Foundation Trust, London SE1 7EH, UK; Randall Centre for Cell and Molecular Biophysics, Muscle Signalling Section, Faculty of Life Sciences and Medicine (FoLSM), King’s College London, London SE1 1YR, UK

**Keywords:** myasthenia gravis, arthrogryposis multiplex congenita, fetal acetylcholine receptor inactivation syndrome, transient neonatal myasthenia gravis, congenital myopathy, salbutamol

## Abstract

*In utero* exposure to maternal antibodies targeting the fetal acetylcholine receptor isoform (fAChR) can impair fetal movement, leading to arthrogryposis multiplex congenita (AMC). Fetal AChR antibodies have also been implicated in apparently rare, milder myopathic presentations termed fetal acetylcholine receptor inactivation syndrome (FARIS). The full spectrum associated with fAChR antibodies is still poorly understood. Moreover, since some mothers have no myasthenic symptoms, the condition is likely underreported, resulting in failure to implement effective preventive strategies.

Here we report clinical and immunological data from a multicentre cohort (*n* = 46 cases) associated with maternal fAChR antibodies, including 29 novel and 17 previously reported with novel follow-up data. Remarkably, in 50% of mothers there was no previously established myasthenia gravis (MG) diagnosis. All mothers (*n* = 30) had AChR antibodies and, when tested, binding to fAChR was often much greater than that to the adult AChR isoform. Offspring death occurred in 11/46 (23.9%) cases, mainly antenatally due to termination of pregnancy prompted by severe AMC (7/46, 15.2%), or during early infancy, mainly from respiratory failure (4/46, 8.7%). Weakness, contractures, bulbar and respiratory involvement were prominent early in life, but improved gradually over time. Facial (25/34; 73.5%) and variable peripheral weakness (14/32; 43.8%), velopharyngeal insufficiency (18/24; 75%) and feeding difficulties (16/36; 44.4%) were the most common sequelae in long-term survivors. Other unexpected features included hearing loss (12/32; 37.5%), diaphragmatic paresis (5/35; 14.3%), CNS involvement (7/40; 17.5%) and pyloric stenosis (3/37; 8.1%). Oral salbutamol used empirically in 16/37 (43.2%) offspring resulted in symptom improvement in 13/16 (81.3%).

Combining our series with all previously published cases, we identified 21/85 mothers treated with variable combinations of immunotherapies (corticosteroids/intravenous immunoglobulin/plasmapheresis) during pregnancy either for maternal MG symptom control (12/21 cases) or for fetal protection (9/21 cases). Compared to untreated pregnancies (64/85), maternal treatment resulted in a significant reduction in offspring deaths (*P* < 0.05) and other complications, with treatment approaches involving intravenous immunoglobulin/ plasmapheresis administered early in pregnancy most effective.

We conclude that presentations due to *in utero* exposure to maternal (fetal) AChR antibodies are more common than currently recognized and may mimic a wide range of neuromuscular disorders. Considering the wide clinical spectrum and likely diversity of underlying mechanisms, we propose ‘fetal acetylcholine receptor antibody-related disorders’ (FARAD) as the most accurate term for these presentations. FARAD is vitally important to recognize, to institute appropriate management strategies for affected offspring and to improve outcomes in future pregnancies. Oral salbutamol is a symptomatic treatment option in survivors.

## Teaser

Allen *et al*. show that *in utero* exposure to maternal antibodies against the foetal AChR may mimic a wide range of persistent neuromuscular disorders in offspring, despite many asymptomatic mothers. Immune-based therapies during pregnancy can be preventative, while oral salbutamol is an effective treatment in offspring.

## Introduction

Myasthenia gravis (MG) is an acquired autoimmune condition of the neuromuscular junction with an incidence of 30/million/year in adults and commonly caused by antibodies against the acetylcholine receptor (AChR). The AChR is a pentameric structure composed of five subunits and exists in a fetal (fAChR) and an adult (adAChR) isoform. Most AChR antibodies bind to both the fetal and adult isoforms, via the shared α, β and δ subunits; however, some MG patients may also have additional antibodies specific to the AChR fetal γ subunit. This subunit is expressed during fetal life but replaced with the adult ɛ subunit to form the adAChR by 33 weeks’ gestation.^1,2^

Transfer of IgG from mother to fetus starts around week 16 of gestation and is maximal by term. Infants born to mothers with MG may either be asymptomatic or, in 10–15%, develop transient neonatal myasthenia gravis (TNMG) characterized by variable generalized weakness, feeding difficulties, respiratory difficulties and facial diplegia. The symptoms of TNMG appear within the first few days of life and mostly resolve by 2 months but may last up to 3 to 4 months in some cases. Rarely, maternal antibodies that bind to the one (of two) acetylcholine binding site adjacent to the fAChR-specific γ subunit, paralyse the baby *in utero* leading to lethal arthrogryposis multiplex congenita (AMC)/fetal akinesia deformation sequence (FADS).^3-6^ Surviving infants may also present with a less severe phenotype with or without AMC, often with marked neonatal hypotonia and a fixed myopathy of variable severity, including a predilection for facial and bulbar muscles.^7-9^ The association between the less severe myopathic-predominant phenotype and the fAChR antibodies was first recognized in 2008 (Oskoui *et al*.^7^) and termed fetal acetylcholine receptor inactivation syndrome (FARIS), and has rarely been reported to date.

It is likely that AMC, FARIS and TNMG are part of a spectrum, with severity and duration of symptoms depending on the specificity of the AChR antibodies and associated pathogenic mechanisms. However, as the number of currently reported cases is only small, the full spectrum of these presentations is likely under-reported.^7,8,10-12^ A few case reports, including our previous series,^8^ suggest that intensive immunomodulatory treatment during pregnancy in MG-affected mothers with previously affected offspring can modify outcomes in future pregnancies, but the optimal mode, timing, intensity and frequency of treatments have not been established yet. Moreover, some mothers may not have been diagnosed with MG at the time of the first affected pregnancies, hampering correct diagnosis in the offspring and the institution of preventative treatment strategies for future pregnancies.^3,4,8,11^ The treatment approach to symptomatic long-term offspring survivors has been mainly supportive, but anecdotal evidence of a beneficial effect of oral salbutamol^9^ suggests a potentially effective pharmacological treatment at least for some symptoms. Owing to the rarity and likely under-recognition of these conditions, numerous questions remain regarding the phenotypic spectrum and natural history as well as the most effective management and preventative strategies.

Here our primary aim was to describe detailed clinical and immunological data and the phenotypic spectrum from the largest cohort of cases associated with maternal antibodies to the AChR identified to date. Our secondary aims were to explore the association between maternally-administered immunotherapies during pregnancy and fetal outcomes, as well as treatment effects of oral salbutamol in affected children. This information will be relevant to neurologists, neonatologists, paediatric neurologists, paediatricians, geneticists and obstetricians looking after myasthenic mothers and their offspring, by providing guidance for the diagnosis, management and prevention of these probably under-reported conditions.

## Materials and methods

### Patients and methods

For our primary aim, we conducted an analytical case series investigation, reporting clinical and immunological data from a multicentre cohort associated with maternal AChR antibodies meeting the inclusion criteria. Novel cases were identified and recruited by clinicians from participating neuromuscular centres, coordinated by N.A. and H.J. as part of an ongoing international collaborative effort.^8,9^ For our secondary aims, we conducted a literature review, and combined data extracted from our cases and historical cases, and applied a retrospective cohort study approach to evaluate the association with maternally-administered immunotherapies during pregnancy, and offspring outcomes. Historical cases were identified from the English and French published literature on MG-related AMC and FARIS. Only cases published between 1972 to 2022 were considered. To obtain longitudinal follow-up data from published cases, we contacted the lead authors.

Primary inclusion criteria were: (i) positivity for maternal antibodies against the fAChR and suggestive clinical features such as AMC and/or other myopathic features persisting >3 months in the index case; (ii) maternal diagnosis of MG with AChR antibodies and suggestive clinical features in the offspring without alternative explanation; or (iii) subsequently born siblings of index cases with similar clinical features. Exclusion criteria were that of alternative (genetic or non-genetic) diagnoses explaining presenting neuromuscular features.

Clinical/phenotypic details were extracted from the medical notes based on a standardized proforma (Supplementary Table 1) and, where possible, verified on a recent clinical assessment. All procedures were conducted as part of routine clinical care. The study was performed under the ethical guidelines issued by the participating institutions for clinical studies. All patients and/or their legal guardians consented to the anonymous publication of their clinical information. Additional written informed consent was obtained from all participants with identifiable photographs.

#### Immunological investigations

Maternal antibody testing was performed on all referred samples mainly by the Oxford Autoimmune Neurology (previously Clinical Neuroimmunology) laboratory and, less frequently, by other specialist centres. All maternal sera were tested for AChR antibody levels using radioimmunoprecipitation. Since radioimmunoprecipitation does not distinguish between the different fAChR and adAChR isoforms, where available, sera were also tested on cell-based assays (CBA) to distinguish binding of serum antibodies to the adult or fetal AChR (as detailed in Hacohen *et al*.^8^). All available maternal sera, and the few sampled from their infants, were tested at serial dilutions and the titres determined from the end-point dilution at which the binding scored 1 (on scale 0–4).

#### Statistical approach

The clinical characteristics of all cases were summarized using descriptive statistics. Median and interquartile range (IQR) were reported for skewed data. To analyse maternally-administered immunotherapies during pregnancy (pIMT) and offspring outcomes, we combined our series with cases from the literature and divided cases not exposed to pIMT and those exposed to any pIMT. Risk ratios with 95% confidence intervals (CIs) were calculated for statistical significance for categorical variables. Independent sample *t*-test and Mann–Whitney U-test were applied for differences in continuous variables. Based on the observations of treatment variabilities and observed outcomes for those receiving more intense immunotherapy regimes, we devised a pIMT score to quantify and compare approaches within the treatment group (Supplementary Tables 2 and 3). SPSS v25 was used for data analysis. The response to neonatal or childhood treatments and outcomes was determined by using a treatment response proportion without a comparator group. Antibody binding scores and ratios were used for fAChR and adAChR antibody investigations.

#### Data availability

Data supporting this study are available from the corresponding authors, upon reasonable request.

## Results

Novel data are presented from 46 offspring (cases) with fAChR-related disorders as well as their mothers (see ‘Current series’ section), including detailed clinical information from 29 unpublished and new follow-up data from 17 previously reported cases.^7,8,11-15^

Combining our series with data from the known literature we identified a total of 89 cases. Longitudinal new data could not be obtained in 43 previously reported (historical) cases (FADS/AMC phenotypes and non-AMC phenotypes), because they were either deceased (*n* = 30)^3,4,8,16-20^ or had been lost to follow-up (*n* = 13).^4,5,8,16,19-27^

**Figure 1:**
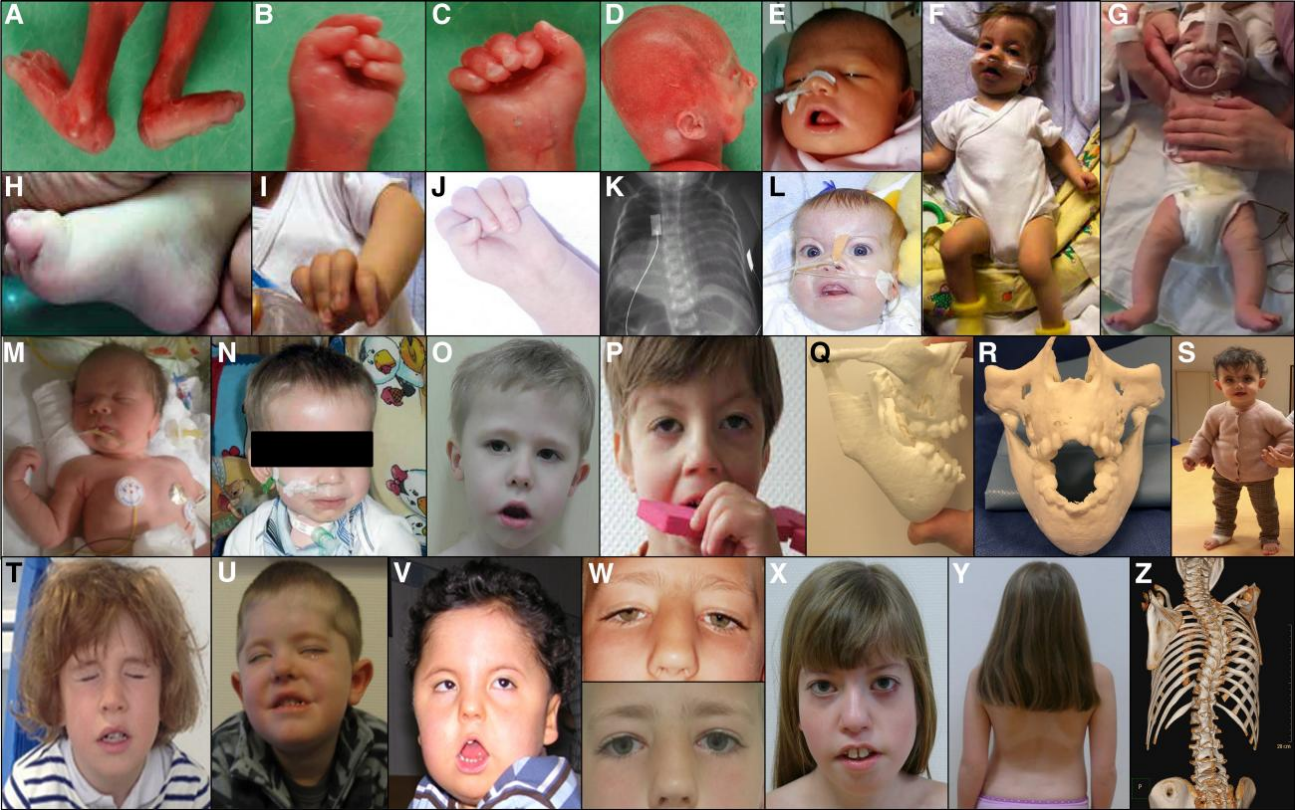
**Clinical features in offspring with fetal acetylcholine receptor antibody-related disorders (FARAD) at different time points**. The offspring of myasthenic mothers with antibodies to the (fetal) acetylcholine receptor has a wide spectrum of features, ranging from lethal arthrogryposis multiplex congenita (AMC) (**A**–**D**) to less severe contracture phenotypes (**F** and **H**–**J**). Congenital hypotonia and facial weakness (**E**) are common. Respiratory impairment requiring (non-invasive) ventilation (**G**) and bulbar involvement requiring nasogastric tube feeds (**F**, **G** and **L**–**N**) are common. Less common features include diaphragmatic paresis (**K**) and extraocular muscle involvement (**L**). Later in life, persistent facial weakness with a myopathic facial appearance (**O**, **P** and **T**–**V,** and **X**), incomplete eye closure and an inverted V-shaped mouth are common. Facial weakness may at least be partially responsive to treatment with oral salbutamol (**W**). Significant jaw contractures (**Q** and **R**, 3D CT reconstruction) in more severely affected patients, and scoliosis (**Y** and **Z**, 3D CT reconstruction) can occur. A small proportion of children may have a longer ventilatory and enteral feeding requirement (**N**).

We identified a spectrum of previously recognized and novel associated features. Maternal, pregnancy, fetal and offspring characteristics of our current series are detailed below and summarized in Table 1. The denominator used for each item reflects available/applicable data only with missing or non-applicable data not included in calculations. Offspring characteristics are illustrated in Fig. 1.

**Table 1:** Clinical characteristics: maternal and fetal, neonatal and infancy, and later follow-up (current series)

Maternal and fetal	*n* = 46	Neonatal-infancy	*n* = 46	Later features	*n* = 46
Families/mothers	30	Hypotonia	35/36 (97.2%)	Facial weakness	25/34 (73.5%)
Single pregnancy only	17	Facial weakness	36/37 (97.3%)	Muscle weakness	14/32 (43.8%)
Pregnancy MG undiagnosed	23/46 (50%)	Craniofacial dysmorphism	24/31 (77.4%)	Jaw contracture/malocclusion	6/32 (18.8%)
Pregnancy MG symptoms	20/41 (48.8%)	Contractures/AMC	31/46 (67.4%)	Extraocular involvement	5/22 (22.7%)
Gestation(w): median; IQR (range)	38; 4 (27–40)	Neonatal intubation	13/37 (35.1%)	Scoliosis	7/44 (15.9%)
Prematurity (<37 weeks)	10/37 (27%)	Neonatal NIPPV	9/37 (24.3%)	Feeding difficulty	16/36 (44.4%)
Antenatal death	7/46 (15.2%)	Pulmonary hypoplasia	10/42 (23.8%)	PEG requirement	8/31 (25.8%)
Antenatal contracture/AMC^a^	12/46 (26.1%)	Diaphragmatic paresis	5/35 (14.3%)	VPI > 1 year	18/24 (75%)
Polyhydramnios	22/43 (51.1%)	Pleural effusion^b^	4/38 (10.5%)	Speech difficulty	22/32 (68.8%)
Reduced fetal movement	11/42 (26.2%)	Pneumothorax	2/36 (5.6%)	Hearing impairment	12/32 (37.5%)
IUGR	7/39 (17.9%)	NGT feeding	33/34 (97.1%)	CNS involvement	7/40 (17.5%)
Maternal treatments	–	Pyloric stenosis	3/37 (8.1%)	Tracheostomy	3/35 (8.6%)
Any IVIG/PLEX/predisolone	15/46 (32.6%)	Cryptorchidism (males)	8/24 (33.3%)	OSA/Restrictive defect	9/28 (32.1%)
Thymectomy^c^	14/46 (30.4%)	Inguinal hernia	7/38 (21.2%)	Resp. support,^d^ days: median (IQR)	45 (85)
AChEi	19/44 (43.2%)	Postnatal death (all infancy)	4/46 (8.7%)	Age at follow-up, years: median (IQR)	7.5 (14)

Percentages are derived from the total number of cases where this feature could be reliably established. AChEi = acetylcholinesterase inhibitor; AMC = arthrogryposis multiplex congenita; IQR = interquartile range; IUGR = intrauterine growth restriction as commented on in publication or if birth weight below 10th centile; MG = myasthenia gravis; NGT = nasogastric tube; NIPPV = non-invasive positive pressure ventilation; OSA = obstructive sleep apnoea; PEG = percutaneous endoscopic gastrostomy; Resp. = respiratory; VPI = velopharyngeal incompetence.

Contractures/AMC category includes a range of cases with contractures in more than one limb up to cases within the severe fetal akinesia deformation spectrum (FADS) presentations.

Includes chylothorax.

Thymectomy performed before the pregnancy.

Includes *n* = 27 infants (excludes Day 1 deaths) (range 2–1825 days).

### Current series

#### Maternal details

##### Maternal histories

A total of 30 families (mothers) were identified. Recurrence in subsequent pregnancies was documented in 13/30 (43.3%) families. Diagnosis of maternal MG had not been established prior to conception in 23/46 (50%) pregnancies. Absence of maternal MG symptoms occurred in 21/41 (51.2%) pregnancies, and was more frequently observed in mothers whose offspring had died [documented in 8/10 (80%) pregnancies] compared to those whose offspring had survived [documented in 13/31 (41.9%) pregnancies]. Where present, maternal MG symptoms were usually mild, with post-partum MG exacerbations reported in 5/41 (12.2%). A personal history of one or more additional common autoimmune disorders was noted in 5/30 (16.7%) mothers.

##### Maternal AChR antibody results

**Figure 2:**
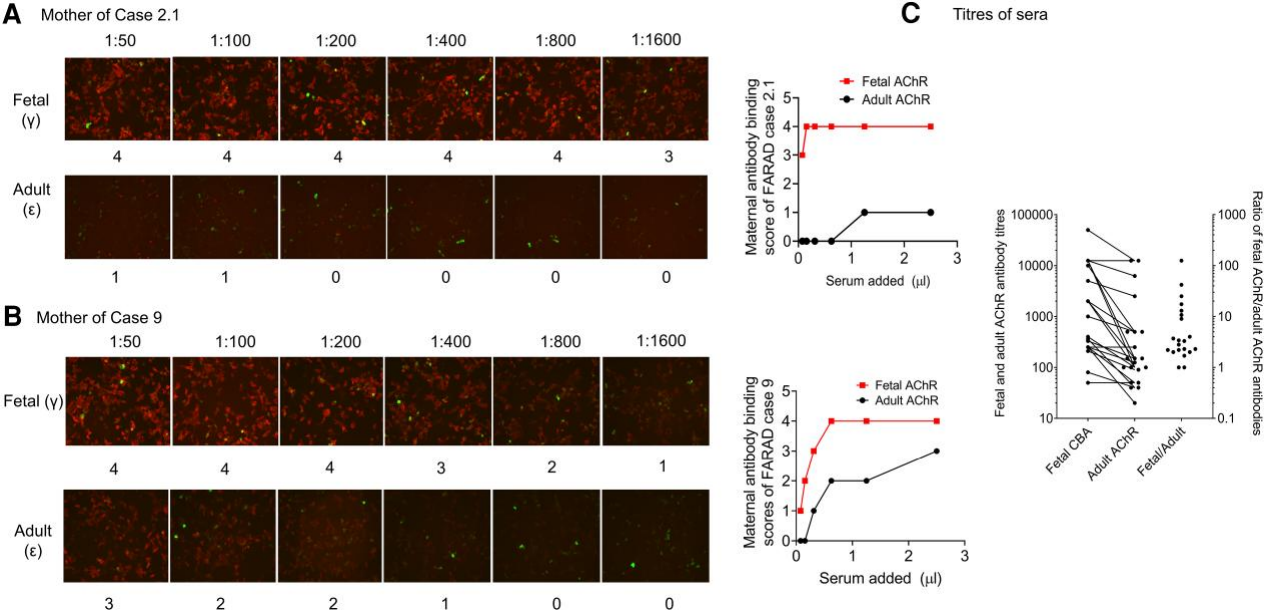
**Maternal acetylcholine receptor antibody results.** Fetal (f) and adult (ad) acetylcholine receptor (AChR) antibody titres varied considerably. The figure shows representative cell-based assays (CBAs) from two mothers with (**A**, Case 2.1) very high fAChR binding scoring 3 even at 1:1600 dilution compared with almost undetectable binding to adAChR at 1:50 dilution; the mother shown in **B** (Case 9) had less fAChR antibodies but more adAChR antibodies than the mother in **A**. Titres of all sera binding to fetal (red) and adult (black) AChRs, tested by CBAs, are shown in **C**, with the ratio of fetal to adult titres illustrating the variability.

Routine maternal AChR antibody titres (usually by radioimmunoassay and measuring antibodies to a mix of adult and fetal AChRs without distinction) were positive in all mothers, with titres ranging from 1.53 to 5240 nM. As illustrated in Fig. 2, CBAs were used to distinguish binding to the native fetal or adult AChRs. Although those assays were generally positive for both isoforms under screening conditions, when sera were serially diluted binding to fAChRs was often, but not invariably, much greater than that to the adAChRs (examples shown in Fig. 2A and B). Accordingly, the end-point dilution titres obtained in the 21 available sera, and the ratio of fAChR/adAChR titres, ranged widely (Fig. 2C).

##### Pregnancy details

Suggestive antenatal signs included features within the AMC spectrum in 12/46 (26.1%), as well as polyhydramnios in 22/43 (51.1%) and reduced fetal movements in 11/42 (26.2%). Median gestation at delivery was 38 weeks (IQR = 4, range 27–40 weeks), with 10/37 (27%) preterm (Table 1). Where documented (36/46 cases), mode of delivery was spontaneous vaginal in 11, Caesarean section in 18 and by termination of pregnancy (TOP) in 7.

#### Offspring details

##### Death and survival in offspring

Twenty-seven (58.7%) cases were male and 19 (41.3%) were female. The number of deaths in our cohort was 11/46 (23.9%). All antenatal deaths [7/46 (15.2%)] were from TOP. Four (8.7%) deaths occurred postnatally, mainly linked to respiratory complications and all during early infancy. Median age at most recent follow-up in the surviving patients (*n* = 38) was 7.5 years ranging from infancy to 46 years (IQR = 14).

##### Neonatal presentations

Growth restriction was present in 7/39 (17.9%) offspring, noted antenatally (as intrauterine growth restriction) or at birth. A head circumference below the 2nd centile was noted in 2/19 (10.5%) neonates.

Feeding difficulties of neonatal onset and due to a combination of sucking and swallowing difficulties were almost universal, with 33/34 (97.1%) cases surviving beyond Day 1 requiring nasogastric feeding, lasting from weeks to several months.

A dysmorphic appearance was noted in 24/31 (77.4%) neonates, based on a combination of myopathic facial features, with a high-arched/narrow palate, micro/retrognathia, low set/prominent ears and, less frequently, hypertelorism and a broad nasal bridge. The combination of intrauterine growth restriction and dysmorphic features often prompted the initial suspicion of a primary genetic/syndromic diagnosis before the autoimmune aetiology was considered, particularly in families where the mother did not have a diagnosis of MG before the pregnancy.

Respiratory involvement was prominent in the neonatal period. Immediate respiratory support was required in 27/37 (73%) cases and included initial oxygen in 5/37 (13.5%), intubation in 13/37 (35.1%) and non-invasive positive pressure ventilation in 9/37 (24.3%) cases. Length of ventilation (invasive or non-invasive) (*n* = 27) ranged from 2 to 1825 days (median 45 days; IQR = 85).

Pulmonary hypoplasia was documented in 10/42 (23.8%) cases, first detected on X-ray in six live-born neonates and on post-mortem in four cases who had died antenatally. Taken together, pulmonary hypoplasia was associated with high mortality, with a total of six deaths in 10 cases. Diaphragmatic paresis (Fig. 1K), a novel association, was observed in 5/35 (14.3%) cases, three of whom required diaphragmatic plication. Two cases of 36 (5.6%) had pneumothoraces, and 4/38 (10.5%) cases had variable effusions (chylothorax/pleural effusion). Neonatal pulmonary hypertension was noted in 7/24 (27.3%) cases, sometimes requiring treatment with inhaled nitric oxide and high frequency oscillatory ventilation.

##### Neuromuscular features

Almost all surviving neonates had generalized congenital hypotonia and weakness [35/36 (97.2%)] pronounced in axial and to a lesser extent limb muscles. Beyond infancy, muscle strength generally improved, with mild residual weakness (Medical Research Council Scale for Muscle Strength score of around 4/5) noted in 14/32 (43.8%) cases. Muscle involvement manifested as motor delay and/or mild gross/fine motor difficulties in younger children and as increased fatigue and/or reduced stamina in older children, mainly without major impact on independent function. Where documented, tendon reflexes could be elicited in most cases. Facial weakness was noted at birth in 36/37 (97.3%) cases, and despite some improvement persisted in 25/34 (73.5%) surviving cases. Extraocular muscle involvement, a novel feature and usually mild, was observed in 5/22 (22.7%) cases, with some improvement over time.

Six of 32 (18.8%) patients had jaw opening deformities, four with jaw open contracture and two with dental malocclusion, causing difficulty with mouth closure, resulting in the recommendation of reconstructive surgery in two cases (Fig. 1Q and R). At least three other cases without definite jaw contractures or documented malocclusion had a persistent tendency to keep their mouths permanently open.

Contractures (both single site and multiple) occurred in 31/46 (67.4%) cases, with diagnostic criteria for AMC (i.e. contractures affecting ≥2 limb regions) fulfilled in 23/47 (51.3%) cases. AMC had been reported antenatally in 12/46 (26.1%) of all pregnancies. Limb contractures, mainly of the flexion type, were more commonly distal [28/45 (62.2%) cases] than proximal [14/45 (31.1%) cases]. Where specified, upper limb contractures [26/44 (59.1%) cases] often affected fingers/wrists/elbows and lower limb contractures [21/40 (53.1%) cases] mainly involved toes/ankles (including talipes equinovarus)/knees/hips. In surviving offspring, there was a tendency for limb contractures to resolve or improve over time.

Scoliosis (usually thoracolumbar and slowly or non-progressive) was detected in 7/44 (15.9%) cases, mainly later in life but congenital in one case who subsequently died. One patient underwent spinal fusion surgery at age 15 years.

##### Bulbar and velopharyngeal involvement

Feeding difficulties frequently persisted beyond the neonatal period in 16/36 (44.4%) cases, with 8/31 (25.8%) requiring percutaneous endoscopic gastrostomy (PEG) insertion. Excluding early deaths and young infants, feeding improved in all cases, with PEG removal possible following a period ranging from 6 months to 10 years in most, except three cases where the PEG was still *in situ* at the most recent review when ages ranged between 4 months and 5 years. Drooling was observed in 15/31 (48.4%) cases, usually improving without medical interventions. Velopharyngeal insufficiency (VPI), either formally documented and/or suspected based on marked (nasal) dysarthria, was documented in 18/24 (75%) cases.

##### Cardiorespiratory features

Respiratory features persisted beyond the neonatal period but tended to improve over time. Tracheostomy was required in 3/35 (8.6%) cases but could be successfully reversed in all. All survivors showed constant improvement of respiratory function, but some had long-term respiratory sequelae. In particular, beyond the neonatal period 9/27 (33.3%) cases were noted to experience more frequent respiratory infections, sometimes associated with aspiration. Intercurrent respiratory infections occasionally resulted in significant acute respiratory deteriorations, with two cases requiring intensive care, one of whom died. Nine of 28 (32.1%) cases had persistent respiratory ventilatory defects, including obstructive sleep apnoea (OSA) and, less frequently, restrictive defects. Of the four post-natal deaths, one occurred on Day 1 of life and three later in infancy, all of whom had experienced respiratory complications including pulmonary hypoplasia and were ventilator-dependent.

In general cardiac involvement was not a feature. One infant had minor congenital heart defects (atrial septal defect and patent ductus arteriosus) and another suffered a transient hypertrophic cardiomyopathy in the setting of rhesus incompatibility.

##### Gastrointestinal, abdominal and urogenital features

Nine of 37 infants (24.3%) experienced persistent vomiting, in the majority due to suspected gastro-oesophageal reflux but in 3 (8.3%) due to a confirmed diagnosis of pyloric stenosis. Inguinal hernias were identified in 7/38 (21.2%) cases. Cryptorchidism was present in 8/24 (33.3%) males.

##### CNS involvement, hearing and vision

Potential CNS involvement was identified in 7/40 (17.5%) cases, all of whom had other significant complications. Problems identified included autism or semantic language disorder (*n* = 3), attention deficit hyperactivity disorder (ADHD) (*n* = 2) or intellectual impairment (*n* = 2). Neuroimaging anomalies of uncertain significance included a thin corpus callosum (*n* = 2), thalamic cyst (*n* = 1) and tethered cord (*n* = 1).

Hearing loss occurred in 12/32 (37.5%) cases and was usually not severe. Six patients had conductive hearing loss (CHL) alone, usually ameliorated with grommet insertion; one patient required insertion of a bone conduction hearing aid. Three cases had isolated sensorineural hearing loss (SNHL), requiring hearing aids. Two patients had mixed CHL and SNHL. Apart from the cases with extraocular movement restriction (5/22, 22.7%) and ptosis there were no visual concerns.

##### Motor and speech development

Motor development was variably delayed in many surviving cases. However, all those followed up beyond infancy achieved the ability to walk, usually in early childhood, without reported disease progression later in life. Normal or near normal motor outcomes were noted when maternally-administered immunotherapies during pregnancy were more intense. Speech difficulties, in particular hypernasality, and most likely due to a combination of bulbar dysfunction, VPI, oromotor co-ordination difficulties and jaw opening contractures, were identified in 22/32 (68.8%) of surviving cases. Despite some improvement over time, speech difficulties tended to persist throughout the follow-up period.

##### Investigations in offspring

Maternally transmitted AChR antibodies were investigated in 17/27 (63%) surviving offspring and detected in 15/27 (negative in two samples sent after age 4 months). Testing for fAChR antibody was only performed in four neonates and positive in all. Many infants, in particular those without a maternal MG history in whom an autoimmune aetiology had not been immediately suspected, underwent extensive additional investigations under the suspicion of an alternative diagnosis, in particular a neuromuscular or neurometabolic disorder (18/22 cases). A range of genetic investigations including amniocentesis karyotypes, chromosomal or single nucleotide polymorphism (SNP) arrays, single gene testing and next generation sequencing (NGS) were performed in 13/17 (76.5%) cases, all normal. Metabolic and infection [in particular, TORCH (toxoplasmosis, rubella, cytomegalovirus, herpes and other agents)] screens were performed and negative throughout. Brain MRIs were performed in seven children and reported as abnormal in two (see above).

EMG was performed in 17/34 (50%) cases, usually during infancy or early childhood, and was reported normal in 7/17 cases. The remainder showed various myopathic (8/17 cases) and, less frequently (2/17), neurogenic features, all without evidence of a neuromuscular transmission defect.

Muscle biopsies taken in 12/38 (31.6%) of cases, during life (*n* = 6) or on post-mortem (*n* = 6), were either normal (*n* = 6) or showed non-specific myopathic features, including type 1 fibre atrophy, variation in fibre size and or fatty infiltration. For a summary of investigations performed in offspring, see Supplementary Table 4.

##### Offspring treatments including salbutamol

Specific treatments in the offspring could be divided into those administered in the neonatal period under the suspicion of TNMG, and those administered later in life to treat chronic (mainly myopathic) sequelae. In 20/35 (57.1%) cases, infants received treatment under an initial suspicion of TNMG. Neonatal acetylcholinesterase inhibitor (AChEi, usually pyridostigmine) was used in 17/34 (50%) cases, at doses of between 2 and 7 mg/kg/day; there was mild and usually transient improvement (of either muscle tone or respiratory function) in five cases. In a few cases, dose escalations were limited by side effects (gastrointestinal, apnoea, bradycardia) after an initial improvement. Intravenous immunoglobulin (IVIG) was administered to 5/36 (13.9%) infants without clear benefit, but neonatal plasmapheresis (PLEX) in 3/34 (8.8%) infants (one mildly affected) was reported to produce a benefit in two.

Based on our previous observation suggesting a potential benefit,^9^ a total of 16/37 (43.2%) additional offspring had been empirically commenced on oral salbutamol (Table 2), with symptom improvement reported in 13/16 (81.3%) offspring. Oral salbutamol was commenced at variable ages ranging from 24 days to 17 years, with doses ranging from 1.5 to 8 mg/day. Responses to oral salbutamol were variable but favorable, including, in decreasing frequency, improved oromotor function, better articulation and increased voice volume, reduced (facial) muscle weakness and reduced fatigue, as well as improved ventilatory requirements (Table 2). In some cases responses were immediate. Side effects, in particular tremor, tachycardia, palpitations and restlessness, limited use in two patients. No benefit was reported in a further patient on moderate doses for 2 months, and in another who gained significant benefit initially but where treatment was discontinued by the parents.

**Table 2:** Effects of oral salbutamol in fetal acetylcholine receptor antibody-affected offspring

Case	Age	Dose	Benefit	Details
1.1	5 years	7.5 mg/d (3–4DD)	Yes	Reduced fatigability and ptosis, mild improvement of articulation and facial expression
3.1	3.5 years	4.5 mg/d (3DD)	Yes	Marked oromotor benefit (tongue mobility, lingual sounds), improved facial weakness (blow, use straw)
4.1	6 months	1.5 mg/d (3DD)	Yes	Improved swallowing, generalized muscle tone, less vomiting
5.1	4 years	6 mg/d (3DD)	Yes	Louder voice
7.3	11 years	8 mg/d (4DD)	Yes	Improved articulation, velopharyngeal symptoms, increased energy
9.1	Neonate	0.5 mg/kg/d (3DD)	Yes	Improved ventilation, tone, limb movements, alertness, reduced apnoea, bradycardia
10.1	3.5 years	3.2 mg/d (3DD)	Yes	Improved oromotor (jaw and tongue) function including chewing but mastication/drinking still slow (normal swallow). As made improvements over subsequent 2 years, parents discontinued it.
12.1	14 years	6 mg/d (2DD)	Yes	Significant sustained improved lower limb fatigability
12.2	8 years	6 mg/d (2DD)	Yes	Sustained improvement in neck and limb fatigability (less than brother Case 12.1)
14.1	12 years	6 mg/d (3DD)	No	Minimal to no self-reported benefit (2 months duration), no side-effects
15.1	4 weeks	0.1 mg/kg/dose (3DD)	Yes	Slight increase in movements, no benefit on ventilation
20.1	10 weeks	3 mg/d (3DD) from 8 weeks	Yes	Initial significant reduction in secretions and choking/aspiration risk (oxygen desaturations and apnoeas); NIPPV wean to self-ventilation
22.1 (F)	17 years	ND	No	Baseline tachycardia and chronic respiratory failure: not a sustained trial
23.2 (F)	5 years	6 mg/d (3DD)	Yes	Improved fatigability, ptosis, facial weakness, oromotor function
28.1 (F)	18 years	4 mg/d	No	Self-discontinued after days due to restlessness, palpitations
29.1 (F)	19 years	4 mg	Yes	Improved musical performance e.g. can now ‘reach the treble with trumpet’, takes salbutamol (4 mg as needed) when going to play with band

Oral salbutamol was prescribed by clinicians on an empirical basis, in offspring at various ages, with variable but sustained benefits in most patients. d = day; DD = divided doses; F = follow-up case; ND = not documented; NIPPV = non-invasive positive pressure ventilation.

Non-pharmacological interventions were applied as required. Palatal elevation devices for VPI were used in two cases. Surgical interventions to address associated complications in some individuals included PEG insertion (*n* = 8), chest drains (*n* = 1), diaphragm plication (*n* = 3), tracheostomy (*n* = 3), orchidopexy (*n* = 7), herniotomy (*n* = 4) pyloromyotomy (*n* = 3), myringotomy-grommets (*n* = 5), ptosis surgery (*n* = 1) and cranial nerve reconstruction for facial weakness (*n* = 3). One 15-year-old patient underwent spinal fusion. Corrective craniofacial surgery is being discussed in two cases. Physiotherapy, speech and language therapy were commonly required. Limb contracture surgeries were not documented (some patients required lower limb orthotics at follow up).

### Maternally-administered immunotherapy during pregnancy and offspring outcome

**Figure 3:**
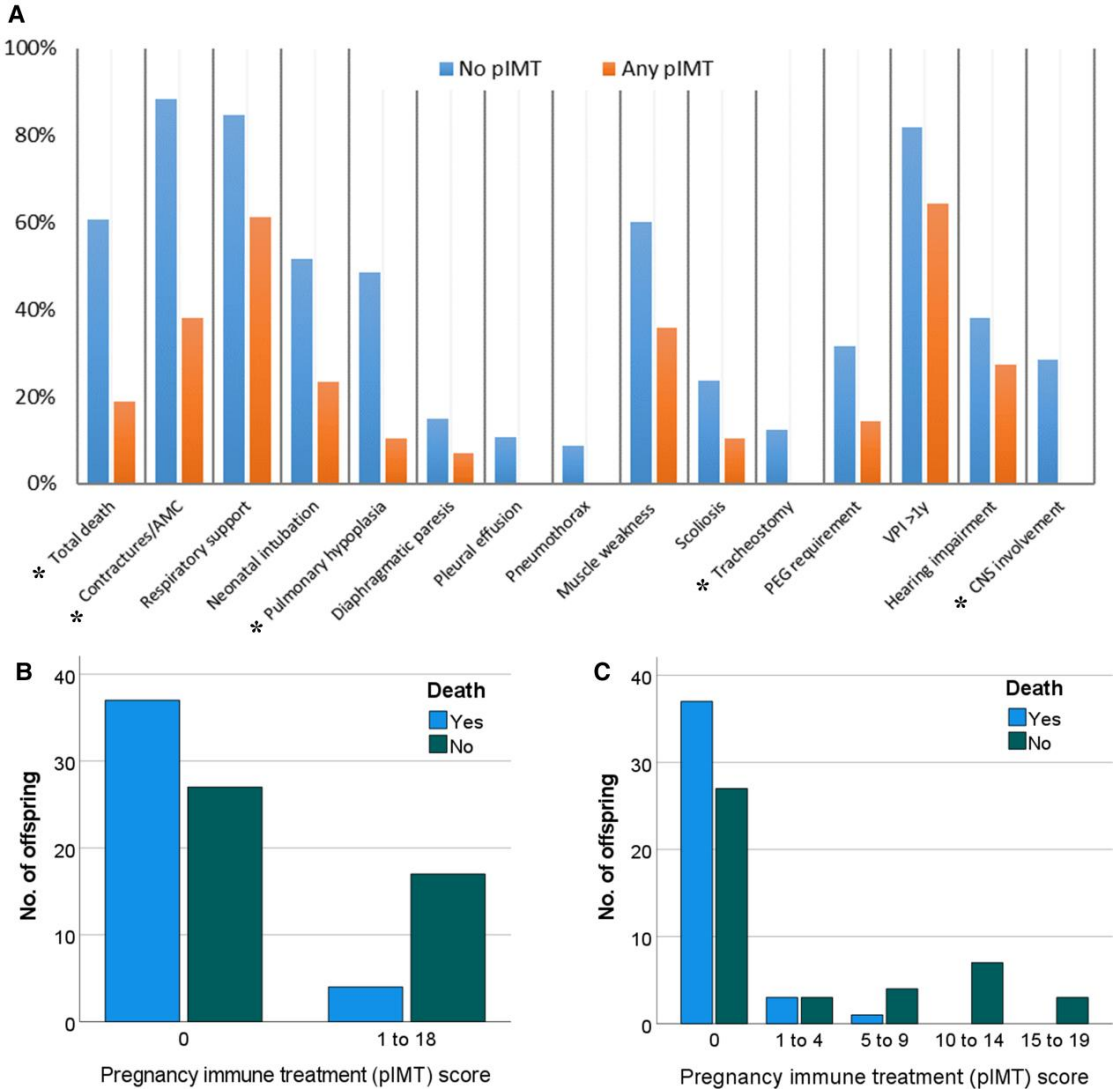
**Correlation between maternal immunotherapy during pregnancy and outcome in offspring.** (**A**) Comparison of overall rate (%) and reduction of major clinical features (complications) in the maternally-administered immunotherapy during pregnancy (pIMT) group compared to those with no pIMT (*P < 0.05). (**B**) Devised score to reflect the mode and timing of treatment during pregnancy (Supplementary Table 3) showing the relative proportion of deaths decreased with the intensity of treatment (*P* < 0.05), and (**C**) no deaths observed with a treatment score above 15. Similar trends were seen for other major features (Table 3 and Supplementary Tables 2 and 5).

We identified 15/46 (32.6%) fetuses where maternal immunotherapy had been administered during pregnancy (pIMT) in our current series, and in addition found 6/39 further pIMT cases from the literature, providing a combined 21/85 (24.7%) cases to compare clinical features and outcomes to 64/85 (75.3%) without pIMT. The pIMT regimes during pregnancy varied by type [single agent or combined use of corticosteroids (15/78; 19.2%), IVIG (7/79; 8.9%) or PLEX (12/79; 15.2%)], intensity (dose and frequency) and timing throughout pregnancies (Tables 1 and 3). Offspring death [relative risk (RR) 3.04, CI 1.38–7.36] and AMC (RR 2.30, CI 1.45–4.23) were more likely in fetuses without pIMT compared to those receiving pIMT; there were also significant differences and reduction trends for the other complications in the pIMT group (Table 3 and Fig. 3A–C).

**Table 3:** Comparing maternal, fetal, neonatal-infancy and later offspring features for no pIMT to pIMT

	Series + literature *n* = 89	pIMT (none) *n* = 64 (75.3%)	pIMT^a^ (any) *n* = 21 (24.7%)	*P*	RR (95% CI)
Maternal-fetal					
Pregnancy MG symptoms	34/66 (51.5%)	22/52 (42.3%)	10/14 (71.4%)	0.03*	0.59 (0.40–1.01)
Gestation, weeks: median; range	38; 27–41	36.4; 27–41	38; 32–40	–	–
Prematurity (<37 weeks)	25/58 (43.1%)	20/40 (50%)	5/18 (27.8%)	0.13	1.80 (0.89–4.14)
Antenatal death	24/89 (27%)	23/64 (56.3%)	1/21 (4.8%)	0.02*	7.55 (1.50–43.03)
Antenatal contracture/AMC	27/81 (33.3%)	24/61 (39.3%)	3/20 (15%)	0.06*	2.62 (1.01–7.71)
Reduced fetal movement	24/68 (35.3%)	18/47 (38.3%)	6/19 (31.6%)	0.60	1.21 (0.62–2.64)
Maternal AChEi	37/78 (47.4%)	18/58 (31%)	17/18 (94.4%)	0.00*	0.33 (0.22–0.50)
Maternal thymectomy^b^	22/82 (26.8%)	9/61 (14.8%)	13/21 (61.9%)	0.00*	0.02 (0.01–0.48)
Offspring death (total)^c^	41/89 (46.1%)	37/64 (57.8%)	4/21 (19%)	0.01*	3.04 (1.38–7.36)
Neonatal-infancy					
Craniofacial dysmorphism	42/51 (82.4)	35/39 (89.7%)	7/12 (58.3%)	0.08*	1.54 (1.07–2.82)
Contractures/AMC^d^	64/87 (73.6%)	56/64 (87.5%)	8/21 (38.1%)	0.00*	2.30 (1.45–4.23)
Respiratory support	41/54 (75.9%)	29/35 (82.9%)	11/18 (61.1%)	0.14	1.36 (0.97–2.17)
Neonatal intubation	20/48 (41.7%)	16/31 (51.6%)	4/17 (23.5%)	0.08	2.19 (0.98–5.58)
Pulmonary hypoplasia	20/58 (34.5%)	18/39 (46.2%)	2/19 (10.5%)	0.02*	4.39 (1.36–16.05)
Diaphragmatic paresis	5/43 (11.6%)	4/29 (14.8%)	1/14 (7.1%)	0.48	1.93 (0.33–12.35)
Pleural effusion^e^	4/55 (7.3%)	4/39 (10.3%)	0/16 (0%)	0.26	3.76 (0.38–38.64)
Pneumothorax	3/53 (5.6%)	3/37 (8.1%)	0/16 (0%)	0.35	3.08 (0.30–32.62)
NGT feeding	38/39 (97.4%)	24/24 (100%)	14/15 (93.3%)	0.54	1.07 (0.91–1.43)
Later features					
Muscle weakness	22/43 (51.2%)	15/27 (55.6%)	5/14 (35.7%)	0.25	1.56 (0.79–3.53)
Jaw contracture/malocclusion	6/40 (15%)	5/27 (18.5%)	1/13 (7.7%)	0.33	2.41 (0.43–14.79)
Scoliosis	15/77 (19.5%)	13/58 (22.4%)	2/19 (10.5%)	0.24	2.12 (0.63–8.05)
Feeding difficulty	24/45 (53.3%)	16/28 (57.1%)	8/17 (47.1%)	0.52	1.21 (0.71–2.29)
PEG requirement	9/36 (25%)	7/21 (33.3%)	2/15 (13.3%)	0.17	2.50 (0.71–9.57)
VPI >1 year	21/29 (72.4%)	10/12 (83.3%)	9/15 (60%)	0.21	1.39 (0.84–2.37)
Speech difficulty	25/38 (65.8%)	14/20 (70%)	9/16 (56.3%)	0.41	1.24 (0.77–2.18)
Hearing impairment	12/33 (36.4%)	9/22 (40.9%)	3/11 (27.3%)	0.43	1.50 (0.59–4.47)
CNS involvement	10/50 (20%)	10/31 (32.3%)	0/15 (0%)	0.04*	10.3 (1.21–100.27)
Tracheostomy	3/44 (6.8%)	3/26 (11.5%)	0/16 (0%)	0.21	4.36 (0.42–45.56)
Resp. support duration^f^, days: median (range)	47 (2–1825)	53 (3–1825)	13 (2–150)	0.05	–

Percentages derive from the total number of cases where this feature was reliably established. AChEi = acetylcholinesterase inhibitor; AMC = arthrogryposis multiplex congenita; IUGR = intrauterine growth restriction; MG = myasthenia gravis; NGT = nasogastric tube; PEG = percutaneous endoscopic gastrostomy; Resp. = respiratory; RR = risk ratios for outcomes based on pIMT (none versus any); VPI = velopharyngeal incompetence. *P < 0.05 considered statistically significant. See Supplementary Table 5 for further details.

pIMT = maternally administered immunotherapy during pregnancy (pIMT not documented in four cases).

Thymectomy performed before the pregnancy.

Includes pre- and postnatal deaths (all postnatal deaths were during infancy).

Contractures/AMC includes a range of cases with contractures in more than one limb up to cases within the severe fetal akinesia deformation spectrum (FADS) presentations.

Includes chylothorax.

Excludes Day 1 deaths.

**Figure 4:**
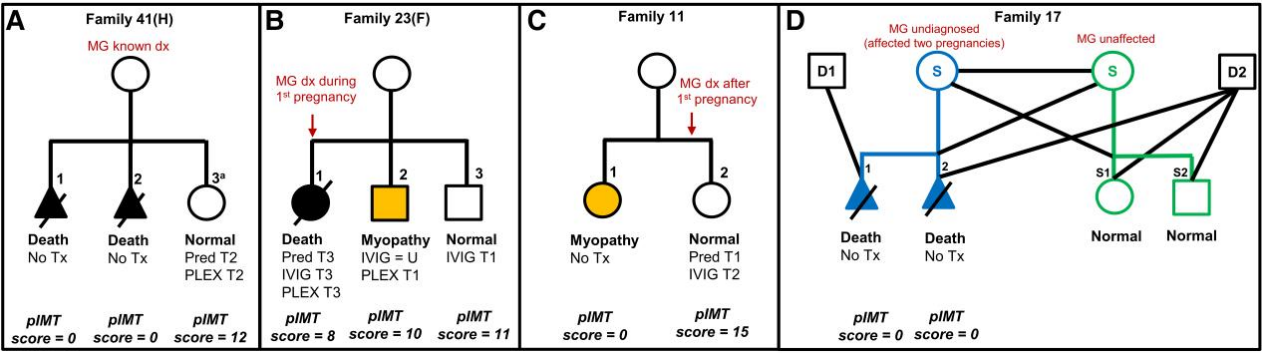
**Illustrative case scenarios showing effect of pregnancy immunotherapy on outcome.** The main determinants of outcome are the mode and timing of immunotherapy during pregnancy, with plasmapheresis (PLEX) and intravenous immunoglobulin (IVIG), alone or in combination with moderate-high dose steroids, early in the pregnancy most effective. Poor outcomes/death seen with less effective (or ineffective) interventions; intermediate outcomes are highlighted in orange; good outcomes are clear. (**A**) Family 41(H) illustrates poor outcome [severe arthrogryposis multiplex congenita (AMC) and death] in two untreated pregnancies, but good outcome in the third pregnancy treated with combination of high dose prednisolone and PLEX in early gestation (^a^TNMG-like but details of longer follow-up not available). (**B**) Family 23(F) illustrates better outcome in the second (orange) and good outcome in the third pregnancies treated with IVIG and PLEX from Trimester 1 onward respectively, compared to the first pregnancy with poor outcome, where similar treatments were administered only from Trimester 3 onwards. (**C**) Family 11 illustrates that the outcome may not be invariably severe even in untreated pregnancies (Case 11.1 with a myopathic non-AMC phenotype following untreated pregnancy) but may still improve further in appropriately treated subsequent pregnancies (Case 11.2, normal at 10 years follow-up). (**D**) Family 17 illustrates a (same-sex) couple who had two previous lethal AMC-affected untreated pregnancies, using ovum from the unaffected (green) partner, to the undiagnosed fAChR antibody-carrying partner/pregnancy (blue), but subsequently used the ovum from the fAChR antibody-carrying partner (blue) for surrogacy to the antibody negative, unaffected partner (green) achieving uncomplicated healthy third and fourth pregnancies (Offspring S1 and S2). D1/D2 = sperm donors; dx = diagnosis; F = follow-up cases, new data; H = historical cases, no new follow-up data; MG = myasthenia gravis; pIMT = pregnancy immunotherapy treatment; Pred. = prednisolone; S = surrogacy; T = trimester; Tx = treatment. See Supplementary Table 2A for all treated cases and dose regimes and Supplementary Table 2B (Table view of Fig. 4).

Based on the observation (particularly within families with recurrent cases) that earlier *in utero* and more intense treatments with single or combined use immunotherapies (IVIG/PLEX/steroids) reduced severity and complications in the offspring, our pIMT score helped quantify the timing, intensity and variability in approaches, for comparisons of outcomes within the pIMT treatment group (Supplementary Tables 2 and 3). As illustrated in Fig. 4, IVIG or PLEX given from the first trimester onward (with or without higher-dose steroid treatment) appeared most effective in preventing complications in the offspring, with a less pronounced or no effect when given late in the pregnancy (see Supplementary Table 2A for complete cases and regimes). Immunotherapies were either administered for maternal MG symptom control (12/21 cases) or for fetal protection (9/21 cases) with less severely affected offspring in the latter group of treated pregnancies, where mean pIMT scores were higher (mean 12.9 versus 5.9; *P* = 0.001) and illustrated in Supplementary Table 2A [Cases 5.2, 11.2, 37.5 (F), 41.3 (H) and 49.4 (H)].

Immunotherapies were administered on the background of a thymectomy performed at variable intervals pre-pregnancy (in 13/21; 61.9% of mothers in the treatment group), and/or treatment with AChEi (17/18; 94.4%) (Supplementary Table 5). AChEi inhibition plus thymectomy without any immunotherapy was used in six cases, two of whom died, two had significant sequelae, one had only finger contractures which resolved (unknown outcome *n* = 1). Two offspring whose mothers had pre-pregnancy thymectomy alone (no AChEi), died. Amongst the offspring of 10 pregnancies where the mothers were treated with AChEi only, six offspring died, three had substantial sequelae of variable severity, and one had a milder TNMG-like phenotype. Azathoprine in combination with prednisolone and PLEX was administered for one pregnancy, following diagnosis of MG, with the offspring showing mild distal finger contractures only^20^ (in the same family, three untreated fetal offspring previously died).^3^

#### Reproductive planning

In our current series, some couples elected not to have further pregnancies due to previous affected offspring and two families opted for surrogacy. One couple who had three previously affected pregnancies, subsequently used their own ovum and sperm and a healthy surrogate mother to have an unaffected child. A same-sex female couple with no symptoms or diagnosis of MG, had two consecutive fatal AMC-affected offspring, where both pregnancies were carried by the same partner, with the other partner as the ova donor. A different sperm donor was introduced for the second pregnancy to reduce risk of a presumed recessive disorder, and the ova donor was investigated (negative for creatine kinase, EMG, AChR antibody titres, and a myopathy gene panel). After the second affected pregnancy, the surrogate mother was investigated with positive EMG and AChR antibodies. The couple pursued further pregnancies with the antibody positive MG-affected partner as the ova donor, achieving uncomplicated healthy third and fourth pregnancies/children (Fig. 4D).

## Discussion

Here we present novel data from 46 cases with a wide range of features secondary to transplacental transfer of maternal antibodies predominantly directed against the fAChR isoform. Our findings suggest a continuous spectrum of features ranging from severe lethal AMC to less severe myopathic manifestations, associated with marked variability in different pregnancies of the same mother, depending on the timing and intensity of maternally-administered immunotherapy during pregnancy. Although the term ‘fetal acetylcholine receptor inactivation syndrome’ (or FARIS) has been previously suggested to describe the milder (non-AMC) end of the disease spectrum, based on our findings we suggest FARAD (or fetal acetylcholine receptor antibody-related disorders) as a more appropriate term, considering the wide clinical spectrum and likely diversity of underlying mechanisms.

Our findings provide novel information regarding the characteristic features and natural history of FARAD and, more importantly, suggest potentially effective therapies and preventative strategies. While most historical (and some of the current) FARAD cases were characterized by severe lethal AMC, our study demonstrates a mild to moderate syndrome characterized by neonatal hypotonia, contractures, bulbar and respiratory muscle involvement often requiring at least temporary ventilatory support and nasogastric tube feeding. Despite often profound symptoms at presentation, there was continuous improvement over time, with sustained improvement into adulthood and good motor function consistently achieved. Residual signs and symptoms included mild weakness and fatigability during exercise and/or intercurrent illness, as well as variable degrees of VPI, dysarthria, dysphagia and, less frequently, persistent respiratory impairment. Intriguingly, some offspring featured additional CNS involvement, diaphragmatic paresis, pyloric stenosis and (both sensorineural and conductive) hearing loss, potentially implicating maternally-transferred (fetal) AChR antibodies also in the evolution of these non-neuromuscular pathologies. The common occurrence of both conductive and sensorineural hearing impairment in our cohort is particularly intriguing and should be investigated in future studies. Conductive hearing loss most likely reflects mechanical features secondary to craniofacial dysmorphism and dysfunction of palatine structures including small muscles (as well as middle ear infections and glue ear), and sensorineural hearing loss likely reflects antibody-mediated direct injury to auditory neurons during development, considering structural similarities between neuronal and muscle AChRs.

The correct diagnosis of FARAD requires a high degree of suspicion, and many affected infants (in particular those of asymptomatic mothers) will initially have been erroneously suspected of having a genetic (neuromuscular) disorder, resulting in often extensive unnecessary investigations. FARAD may mimic early-onset congenital myopathies, congenital myasthenic syndromes, congenital myotonic dystrophy, Prader–Willi syndrome, Moebius syndrome and VPI, and should therefore be considered in the differential diagnosis of these conditions. Despite major recent advances in NGS diagnostics, a substantial proportion of cases of congenital neuromuscular disorders remains currently unresolved, and although the majority of such cases will have an undiscovered genetic aetiology,^28^ our study also raises the possibility of FARAD as an important differential diagnosis in a proportion of such cases. FARAD is also an important consideration in symptomatic offspring of myasthenic mothers initially diagnosed with ‘TNMG’ where symptoms persist beyond the expected 4 months of age.

Bearing in mind these observations, we suggest that FARAD should be considered in the differential diagnosis of all children presenting with suggestive features (particularly a combination of hypotonia, weakness, joint contractures, craniofacial dysmorphism, VPI and respiratory insufficiency), and that appropriate testing instigated without further delay, to inform counselling and prevent potentially devastating disease in future pregnancies. The first step to diagnosis (as in MG) is routine maternal AChR antibody testing, as these test summarily for antibodies against both adult and fAChR isoforms; experience indicates that these antibodies remain positive post-partum for prolonged periods even in clinically asymptomatic mothers. Cord blood or available early neonatal blood will closely reflect maternal levels, but neonatal levels typically drop fast, and maternally transferred AChR antibodies may not be detectable after the first few months of life. If maternal AChR antibodies are positive, discrimination between adult and fetal isoforms requires titrating the sera against cell-based assays, currently mainly done in research laboratories.

A striking observation in our cohort was the beneficial effect of daily oral salbutamol treatment, prompted by its recognized efficacy in our original patient,^9^ and in this study a further 13/16 affected offspring. The improvement was variable but consistent, and typically of rapid onset, including specific improvements of oromotor and bulbar function, ptosis and facial weakness, fatigue and muscle strength and, in one preterm infant, respiratory function. Although the precise mechanism is unknown, these observations are in keeping with the notion that oral salbutamol is effective in a subset of genetic congenital myasthenic syndromes (CMS), in particular *DOK7*- and *COLQ*-related CMS, and has been demonstrated to improve AChR cluster assembly and neuromuscular junction architecture in a mouse model of MuSK-antibody MG.^29^ The mechanism of salbutamol in our cohort may resemble its mechanism in CMS, also considering that other, less-specific adrenergic agonists have been successful in some instances of MG.^30,31^

A substantial number of intrauterine deaths, typically due to TOP (prompted by severe AMC), were recorded in the offspring of mothers where MG had not been suspected and who had therefore not received any immunotherapy before or during pregnancy. At the other end of the spectrum (including some of the same mothers during different pregnancies), offspring survival improved significantly, and the severity and rate of complications reduced in those mothers who received immunotherapy during pregnancy, in particular early and regular IVIG and/or PLEX, with or without higher prednisolone doses (Supplementary Table 2A). Despite a high degree of variability of offspring features as well as the type, timing and intensity of immunotherapies during pregnancy, we were still able to draw a number of conclusions, also reflected in the correlation between offspring outcomes and a specifically-devised pregnancy immunotherapy (pIMT) score. In particular, we observed a greater degree of improvement in those offspring of mothers receiving more intensive and better-timed treatments, with dramatic benefits particularly noted between treated and untreated cases in the same families (as illustrated in Fig. 4 and Supplementary Table 2A). On the other hand, many offspring where the mother had received less intense treatments administered late in the pregnancy suffered more significant sequelae. In this context, it is also noteworthy that in 12/21 treated cases, immunotherapy was administered mainly for maternal MG symptom control, whereas in 9/21 treated cases, immunotherapy was administered specifically for fetal protection, prompted either by a previous affected sibling or by suggestive antenatal signs. In the latter group the average pIMT score was twice as high, and except for one outlier (Case 36.7H, TOP at 19 weeks in association with a low score and established AMC), outcomes were better, with a combination of immunomodulation (IVIG and/or PLEX) and immunosuppression (corticosteroids) early in the pregnancy leading to apparently normal or near normal outcomes in specific cases (Supplementary Table 2A). However, lack of details regarding precise dose regimes and outcomes in some of the historical cases hamper the formulation of clearer within-treatment group comparisons and the most ideal and safest preventive strategies. Lastly, we also noted that a larger proportion of mothers in the treated group were on regular AChE inhibition or had a thymectomy performed prior to pregnancy; it is uncertain if in particular the latter may have had an additional protective effect on the fetus.

Our findings suggest that in families with recurrent unexplained fetal akinesia, or other signs of FARAD, a high degree of suspicion should prompt screening of maternal (fetal) AChR antibodies to identify this presumably rare but probably under-reported and, most importantly, treatable condition. Where a diagnosis of FARAD is established before or during early pregnancy, expert multidisciplinary planning is required for each individual family to achieve optimal *in utero* fetal protection, involving a neurologist experienced in the management of maternal MG, an obstetrician, a neonatologist, a paediatric neurologist and other members of the multidisciplinary team. Delivery of the neonate should ideally occur in an advanced level neonatal unit. Where the mother is symptomatic, MG treatment should be optimized well before the pregnancy, with additional interventions aimed at protecting the fetus using maternally administered immunotherapies during pregnancy. Close monitoring of the pregnancy is paramount, bearing in mind that in some pregnancies fetal involvement may be subtle, despite significant sequelae occurring postnatally (Morel *et al*.^12^ Jeannet *et al*.^13^ and Case 11.1). Whilst on the basis of the currently available evidence (Class IV) it is not possible to provide more detailed treatment recommendations, general principles of treatment should include regular immunomodulation (with IVIG, PLEX) on the background of optimized steroid treatment (particularly in symptomatic mothers), beginning in the first trimester well-before before maternal transfer of IgG antibodies becomes established and extending at least until 33 weeks’ of gestation under close monitoring of the pregnancy. In each case, potential benefits for the fetus have to be carefully weighed against the risks of treatments to both the mother and fetus. Monitoring for signs of fetal disease by conventional approaches such as standard antenatal ultrasound may fail to detect fetal complications such as AMC/contractures and therefore a fetal medicine approach (including fetal MRI) may be appropriate.

Although their principal involvement is now generally accepted, there is some uncertainty concerning the specific mechanisms by which the maternal (fetal) AChR antibodies result in AMC and a permanent but slowly improving myopathy rather than merely the TNMG which has been recognized for 80 years.^32^ TNMG is presumably linked to maternal antibodies against the adAChR isoform, although most studies focusing on TNMG have not distinguished between specific AChR antibody subtypes. In a small number of AMC cases studied, the maternal antibodies were shown to directly block the function of the AChR, paralysing the fetus during the second trimester.^6,33^ These antibodies also resulted in AMC when injected into pregnant mice.^34^ It is possible that the antibodies in mothers whose babies survive do not completely paralyse the offspring but also act by reducing the numbers of AChRs, as they do in MG, thus explaining the much less severe intrauterine findings. Postnatally, as the baby begins to make complement, complement-mediated damage might occur as described in one case.^11^ Nevertheless, these mechanisms would not easily explain the myopathic features in the surviving offspring in the absence of EMG evidence of neuromuscular junction defect.

Irrespective of the precise pathogenesis, it is of note that the severe end of the FARAD spectrum particularly, shares many features with Escobar syndrome due to inactivating recessive *CHRNG* mutations affecting the AChR fetal γ subunit,^35^ emphasizing the crucial role of the fAChR receptor in both presentations. In particular, features shared between *CHRNG*-related Escobar syndrome and FARAD include AMC, craniofacial dysmorphism and weakness, high-arched palate and, variably, respiratory distress, scoliosis, cryptorchidism in males and, interestingly, hearing loss.^36^ Multiple pterygia, one of the defining features of *CHRNG*-related Escobar syndrome, are, however, not typically seen in FARAD, probably reflecting the disproportionate severity of the former. Moreover, neither *CHRNG*-related Escobar syndrome nor FARAD feature neurophysiological evidence of an active neuromuscular transmission defect or respond to AChEi; these commonalities emphasize that the pathology in FARAD has occurred antenatally and is not ongoing, helping to distinguish it from other AChR-related genetic and autoimmune disorders. The notion of predominantly antenatal onset disorders is also supported by findings in mice where the fetal AChR subunit was demonstrated to be prominently and developmentally expressed in head, paravertebral and trunk muscles,^35^ a pattern corresponding to the major sites of involvement in both *CHRNG*-related Escobar syndrome and FARAD. Finally, whilst most findings in our cohort unequivocally suggest the notion of a persistent myopathy, some of the early infancy/neonatal features (for example hypotonia, respiratory and feeding difficulties) could in theory also reflect overlapping mechanisms due to antibodies against the adAChR isoform as seen with typical TNMG phenotypes. This suggestion is also in keeping with an, albeit limited and transient, anecdotal response to AChEi in 5/34 offspring in the current series. Ultimately, more detailed *in vitro* studies on the maternal sera, and more detailed evaluation or adaptation of the mouse models of autoimmune mediated AMC^34^ and Escobar syndrome,^35^ should help to elucidate the precise mechanisms involved in the clinical spectrum of FARAD.

This comprehensive multicenter series of an apparently rare condition emphasizes the complex relationship between maternal AChR antibodies and the increasingly recognized wider spectrum of FARAD. Limitations of this study include the problems inherent to all retrospective studies, the incomplete access to historical and longitudinal data despite intense efforts to obtain those, reflecting the fact that many cases had died and that some had been lost to follow-up. Although the description of many clinical features was based on a recent assessment of mainly paediatric patients by individual participating clinicians, maternal and fetal data were often less complete, in particular in mothers where MG had not been diagnosed at the outset of the pregnancy. Moreover, whilst our study accurately reflects the outcomes of the limited number of fetuses, offspring and mothers included, the heterogeneity of treatment approaches both in terms of modalities and timing reduces power to detect statistically significant differences between treated and untreated groups as evidenced by wider confidence intervals for some features; nevertheless, our data collection provides a framework for future data collection and treatment considerations. Finally, there is also the risk of ascertainment bias in our series towards the more severe end of the spectrum, considering that in more mildly affected children of asymptomatic mothers an autoimmune aetiology may never have been suspected.

In conclusion, where suggestive features are present during gestation or postnatally, FARAD should be considered in fetuses and children of mothers with overt MG but also those who do not have overt myasthenic symptoms. The first step to diagnosis is routine maternal AChR antibody testing (as for MG). Early consideration of these probably under-recognized conditions is important, not only to institute effective preventative strategies but also to avoid the erroneous suspicion of an underlying genetic condition with potentially adverse consequences for family counselling and reproductive planning. Early immunotherapy regimes may improve outcome in future pregnancies, but important questions remain regarding optimal timing, frequency and intensity of these interventions. Salbutamol may be an effective treatment option in offspring with this otherwise untreatable condition.

## Supplementary Material

awad153_Supplementary_Data
