# Supplementary material for: The emerging spectrum of fetal acetylcholine receptor antibody-related disorders (FARAD)
Source: Brain. 2023 May 15;146(10):4233–46. doi: 10.1093/brain/awad153 (PMC10545502; doi:10.1093/brain/awad153)
Supplement: awad153_Supplementary_Data [file awad153_supplementary_data.pdf]

Supplementary Table 1. Data collection sheet

Maternal history / data

1) History of maternal MG (Y/N) any details?
2) Details of maternal treatment prior/during pregnancy (as precise as possible)
3) Maternal treatment prior to suspicion
4) Maternal symptoms pre during post pregnancy
5) History of previous pregnancy/miscarriages and details?
6) Decision not to have further children
7) (Family) history of other autoimmune disorders
8) Oligo/polyhydramnios when etc
9) Fetal movements/ in utero AMC contractures etc etc
10) AChR results

Infant

1) Demographics
    a. Gender
    b. Age at last follow-up
2) Perinatal history
    a. IUGR
    b. Birth measurements height, weight and head size
    c. Apgar indices
    d. Details of resuscitation
    e. Details of neonatal examination
        i. "Dysmorphism" (low set ears, ptosis, small chin, high arch palate, etc etc
        ii. Neonatal neurological exam
3) Development anything relevant and course
    a. Motor development
    b. Speech development
    c. Hearing
    d. Vision
4) Respiratory involvement
    a. Ventilatory support (details and length of)
    b. Pulmonary complications (e.g. diaphragmatic hernia, pulmonary hypoplasia)
5) Bulbar involvement
    a. NG tube feeding (details and length of)
    b. Gastrostomy feeding (details and length of)
    c. Dysarthria
    d. Dysphagia
6) Other medical problems
    a. Pyloric stenosis
    b. Diaphragmatic hernia
    c. Hearing loss (conductive, sensorineural)
    d. Learning difficulties, autism
    e. Others
7) Examination findings
    a. Weakness (facial, axial, limbs)
    b. Ptosis
    c. EOM involvement
    d. Contractures (distribution and course)
    e. Scoliosis
    f. Details of surgical interventions (if any)
    g. Ears
    h. Chin
8) Investigations
    a. Antibodies (age done in mother and infant)
    b. EMG/NCS
    c. Genetics
    d. Muscle biopsy
9) Natural history
    a. Age at last follow-up
    b. Duration of follow-up
    c. Description of disease trajectory (improving, stable, deteriorating)
    d. Typical residual findings at last follow-up
    e. Relationship between maternal treatment and outcome
10) Response to treatment
    a. Pyridostigmine
    b. Salbutamol
    c. IVIG/Plasmapheresis
    d. Anecdotal observations

Anything else relevant interesting about your case please do include

Supplementary Table 2a. Maternal therapies including pregnancy immunotherapies (pIMT)(intensity and timing) and effect on offspring

Case/ Reference	Prednisolone		IVIG		PLEX		pIMT Score	Maternal AChEi	Thym-ectomy	Offspring outcome			Comment for other pregnancies
	Regime	Trim.	Regime	Trim.	Regime	Trim				Dead	AMC	Other	
5.2*	q.o.d: 50mg pre-natal to 5m; 30mg at 5m until last 7m then 10mg	1	6-weekly from T2	2	6 courses	2	18	Y	Y	N	N	9m: minimal issue, normal development	No therapy, significant sequelae in a sibling
9.1*	"low dose" until 23w, then q.o.d x 1m, then 20mg/d	1	At 23w (x 5d) and 27w	2	x5d: at 23w and 4w later	2	16	Y	N	N	Y	8w: 32w gestation, NIPPV, distal contractures, NG fed, improving	N/A
10.1**	15mg/d	1	N	N/A	N/A	N/A	3	Y	N	N	N	18m: orofacial weakness, dysphagia (improving)	N/A
11.2*	Low dose pre/increased early pregnancy (5 to 20mg/d)	1	Monthly from m4 to m9	2	N/A	N/A	15	Y	Y	N	N	10y: normal	No therapy, significant sequelae in a sibling
12.1**	35mg/d continuously	1	N	N/A	N/A	N/A	9	N	Y	N	N	11y: Facial weakness, mild limb fatigability	13.2 sibling below, lower pred. dose, more severely affected
12.2**	10mg/d x 10 wks, then 15mg/d	1	N	N/A	N/A	N/A	4	N	Y	N	N	5y: fatigable, facial weakness, nasal	13.1 above sibling better outcome with higher pred. dose
14.1**	40mg q.o.d	1	N	N/A	N/A	N/A	6	Y	N	N	Y	12y: mild face, axial weakness, distal contractures, nasal	N/A
22.2 (F) [Hacohen *et al* 1.2)]**	N	N/A1	N	N/A	q.o.d x 3w end T2	2	6	U	N	N	Y	4y: "less affected than older sister", nasal speech	Previous sibling no therapy, more severe phenotype
23.1 (F) [Hacohen *et al* 2.1]**	At 27w, 10 to 70mg/d	3	Course at 27w	3	At 20w x 8 cycles	2	8	N	N	Y	Y	Died, AMC, pulmonary hypoplasia	Subsequent offspring less affected with intense therapy
23.2 (F) [Hacohen *et al* 2.2]**	N	N/A	As required	U	x3 near T2	1	10	Y	Y	N	N	6y: less affected (prior sibling died); but weak face, mild motor, speech	See 23.1 (F): severe (died)
23.3 (F) [Hacohen *et al* 2.3]**	N	N/A	From m1 to m5	1	N/A	N/A	11	Y	Y	N	N	5y: markedly less severe than Sibling F2, completely resolved after infancy	See 23.2 (F): IVIG was ad hoc.
24.1 (H) [Hacohen *et al* 3.1]**	20mg q.o.d from outset	1	N	N/A	N/A	N/A	4	Y	Y	Y	U	Died at 17 days respiratory failure	Yes, below sibling survived
24.2 (H) [Hacohen *et al* 3.2]**	10mg q.o.d	1	N	N/A	N/A	N/A	4	N	Y	N	U	Facial and bulbar weakness	see 24.1 (H), died
25.1 (H) [Hacohen *et al* 4.1]**	N	N/A	N	N/A	At 27w, x7 exchanges	3	3	Y	N	Y	Y	5m: died	N/A

28.1 (F) [Oskoui *et al* sibling 1]**	Yes, unknown intensity	U	N	N/A	N/A	N/A	3	Y	Y	N	N	18y: speech difficulty/ facial weakness	Subsequent pregnancies treated more intensely
28.2 (F) [Oskoui *et al* sibling 2]*	Yes, unknown intensity	1	N	N/A	Bi-weekly after T1	2	11	Y	Y	N	N	16y: less severe compared to 1st sib. speech/ face weakness	Previous sibling more affected, less intense regime
28.3 (F) [Oskoui *et al* sibling 3]*	Yes, unknown intensity	1	N	N/A	Bi-weekly throughout	1	14	Y	Y	N	N	15y: less severe compared to 1st and 2nd sibs, facial weakness	Previous 2 siblings more affected, less intense regime
36.7 (H) [Brueton *et al* sibling II-7]*	N	N	N	N/A	5d course at 12w and 16w	2	4	Y	N	Y	Y	TOP at 19 weeks, no conclusion	5 previous related deaths
37.5 (F) [Polizzi *et al*: sibling 6 of Barnes et al]*	Y, unknown intensity (+AZT unknown dose)	U	N	N/A	Yes, unknown intensity	U	12	U	Y	N	N	5th finger contracture only	4 previous affected; 3 deaths, 1 survivor sequelae
41.3 (H) [Carr *et al*: third child)]*	Yes 40mg/80mg q.o.d	2	N	N/A	x6, w5-17	2	14	U	N	N	N	Excellent, TNMG-like phenotype only	2 prior pregnancies lethal FADS (no/unknown regimes)
49.4 (H) [Polizzi *et al:* Newsom-Davis P4]*	U	N/A	Yes, unknown	U	Yes, unknown intensity	U	12	U	Y	N	N	"normal"	3 previous related-deaths

Notes: Full outcome indeterminate in some (e.g. case 10.1 still an infant improving). *Cases (n=9/21) where immunotherapies (pIMT) were mainly targeted toward fetal protection (previous sibling affected or obstetric signs) and in most cases not in early pregnancy; **Cases (n=12/21) in which pIMT were administered mainly for maternal MG symptoms. The mean pIMT score for the "fetal protection" treatment group was 12.9 versus the "maternal MG symptoms" treatment group of 5.9 (p=0.001; 95% CI 3.6-10.3; independent sample t-test). There is a greater proportion of better outcomes [Cases 5.2, 11.2, 37.5 (F), 41.3 (H), and 49.4 (H)] in the treatment toward "fetal protection" group [except for outlier (Case 36.7H) which was a termination of pregnancy at 19 weeks in association with a lower individual score]. **Abbreviations:** (F) = case with follow up data; (H) = case with historical data from publication only; PLEX = plasma exchange; alt = alternate; IVIG = intravenous immunoglobulin; FADS = fetal akinesia deformation sequence; NG = nasogastric feeding; TOP = termination of pregnancy; d = days; w = weeks, m = months; y = years; q.o.d. = every other day; U = unknown; sib = sibling; Trim (T) = trimester; NIPPV = non-invasive positive pressure ventilation; AMC = arthrogryposis multiplex congenital; P4=patient 4 of that reference.

References to Supplementary Table 2

Barnes PR, Kanabar DJ, Brueton L, et al. Recurrent congenital arthrogryposis leading to a diagnosis of myasthenia gravis in an initially asymptomatic mother. Neuromuscular disorders 1995;5(1):59-65.

Brueton LA, Huson SM, Cox PM, et al. Asymptomatic maternal myasthenia as a cause of the Pena-Shokeir phenotype. American journal of medical genetics. 2000;92(1):1-6.

Oskoui M, Jacobson L, Chung WK, et al. Fetal acetylcholine receptor inactivation syndrome and maternal myasthenia gravis. Neurology. 2008;71(24):2010-2012.

Hacohen Y, Jacobson LW, Byrne S, et al. Fetal acetylcholine receptor inactivation syndrome: A myopathy due to maternal antibodies. Neurology(R) neuroimmunology & neuroinflammation. 2015;2(1):e57.

Carr SR, Gilchrist JM, Abuelo DN, Clark D. Treatment of antenatal myasthenia gravis. Obstetrics and gynecology. 1991;78(3 Pt 2):485-489.

Polizzi A, Huson SM, Vincent A. Teratogen update: maternal myasthenia gravis as a cause of congenital arthrogryposis. Teratology. 2000;62(5):332-341.

Supplemental Table 2b. Illustrative case scenarios showing effect of pregnancy immunotherapy (pIMT) on outcome

Case/ family	Intervention							Outcome			
	Pred.	T	IVIG	T	PLEX	T	pIMT Score	Survival	Severe AMC	Myop.	Norm.
**41.1 (H)**	N	-	N	-	N	-	0	Dead	Y		
**41.2 (H)**	N	-	N	-	N	-	0	Dead	Y		
**41.3 (H)**	Y	2	N	-	Y	2	12	Alive			[a]Y
**23.1 (F)**	Y	3	Y	3	Y	3	8	Dead	Y		
**23.2 (F)**	N	-	Y	U	Y	1	10	Alive		Y	
**23.3 (F)**	N	-	Y	1	N	-	11	Alive			Y
**11.1**	N		N		N	-	0	Alive		Y	
**11.2**	Y	1	Y	2	N	-	15	Alive			Y
**17.1**	N	-	N	-	N	-	0	Dead	Y		
**17.2**	N	-	N	-	N	-	0	Dead	Y		
**17-S**	←Surrogacy→							Alive			Y
**17-S**	←Surrogacy→							Alive			Y

The main determinants of outcome are the mode and timing of immunotherapy during pregnancy, with plasmapheresis (PLEX) and intravenous immunoglobulin (IVIG), alone or in combination with moderate-high dose steroids, early in the pregnancy most effective. Poor outcomes due to less effective (or ineffective) interventions are highlighted by dark grey shades, intermediate outcomes are highlighted by light grey shades, good outcomes are not shaded. Family 41 illustrates poor outcome (severe AMC and death) in two untreated pregnancies, but good outcome in the 3rd pregnancy treated with combination of high dose prednisolone and PLEX in early gestation ([a]TNMG-like but details of longer follow up not available). Family 23 illustrates better outcome in the 2nd and good outcome in the 3rd pregnancies treated with PLEX and IVIG from trimester 1 respectively, compared to the 1st pregnancy with poor outcome where similar treatments were administered only from trimester 3 onward. Family 11 illustrates that the outcome may not be invariably severe even in untreated pregnancies (Case 11.1 with a myopathic non-AMC phenotype following untreated pregnancy) but may still improve further in appropriately treated subsequent pregnancies (Case 11.2, normal at 10 years follow -up). Family 17 illustrates a (same-sex) couple who had two previously affected lethal AMC affected untreated pregnancies, but subsequently used their own ovum for surrogacy (to the antibody negative, unaffected partner) achieving uncomplicated healthy 3rd and 4th pregnancies and offspring (cases 17-S). H = historical cases; F = follow up cases; Pred. = prednisolone; AMC = arthrogryposis multiplex congenital; Myop. = myopathic phenotype; Norm. = Normal; Y= yes. See Supplementary Table 2a for all treated cases and dose regimes.

Supplementary Table 3. Pregnancy immunotherapy (pIMT) score derivation

	Score	T1	T2	T3
**IVIG**	= 3	(x3)	(x2)	(x0.5)
**PLEX**	= 3	(x3)	(x2)	(x0.5)
**High dose (>40-80mg/day) steroids***	= 3	(x3)	(x2)	(x0.5)
[IVIG/PLEX not deemed regular/repeated/intense]	= 2	(x3)	(x2)	(x0.5)
[unknown gestation = 6 only]				
[+2 if given across two trimesters beginning <20 wks]				
**Moderate steroids (20-40mg/day)**	= 2	(x3)	(x2)	(x0.5)
(unknown gestation = 4 only)				
(+2 if given across two trimesters as above)				
**Lower steroids (<20mg/day)**	= 1	(x3)	(x2)	(x0.5)
(unknown gestation = 2 only)				
(+1 if given across trimesters as above)				
If unknown steroid dose and gestation then 3 only.				
	**Max = 29**			

This pIMT score calculation is not intended as treatment recommendation, rather to quantify timing, intensity and variability in treatments. The main determinants of scoring were the mode and timing of treatment during pregnancy, with combinations of intravenous immunoglobulin (IVIG), plasmapheresis (PLEX) and high dose steroids given early in the pregnancy attributed higher score, and seen as most effective. Scores are additive for steroids, IVIG and PLEX. *Steroid categorisation (scoring): high-moderate-lower dose was based on increased likelihood of immunosuppression, and is not intended as a treatment guideline. Other immunosuppressant included only 1 patient receiving azathioprine. T = trimester (T1 up to 13 weeks; T2 from 13 to 26 weeks; T3 from 27 to 40 weeks).

Supplementary Table 4. Treatments administered and investigations performed in offspring of myasthenia gravis/AChR antibody positive mothers

	**Current series**	**NA/ND**	**Literature**	**NA/ND**
**Cases**	**46**		**89**	
**Offspring investigations**				
EMG	17/34 (50%)	12	23/50 (46%)	39
Muscle histology (biopsy/pm)	12/38 (31.6%)	8	24/61 (39.3%)	28
Genetic investigations	13/17 (76.5%)	29	14/18 (80%)	71
**Offspring treatments**				
Any Treatment	20/35 (57.1%)	11	29/58 (50%)	31
AChEi	17/34 (50%)	12	25/56 (44.6%)	33
IVIG	5/36 (13.9%)	10	5/58 (8.6%)	31
PLEX/ET	3/34 (8.8%)	12	4/56 (7.1%)	33
Salbutamol	16/37 (43.2%)	9	16/51 (31.4%)	38

Treatments (except salbutamol) were given in the neonatal period or early infancy under the suspicion of Transient Neonatal Myasthenia Gravis (TNMG). Salbutamol was administered to some neonates but at later ages of follow up in the majority. ND = no data; NA = not applicable; AChEi = Acetylcholine esterase inhibitors; EMG = electromyography; ET = exchange transfusion; pm = post mortem

Supplementary Table 5. Maternal, fetal, neonatal-infancy and follow up clinical characteristics comparing outcomes for no pIMT to pIMT

Features	Current series	Current series + literature review	pIMT (none)	pIMT[a] (any)	*P*	RR	CI	IVIG/PLEX (no steroids)
	**n=46**	**n=89**	**64/85 (75.3%)**	**21/85 (24.7%)**				**6/21 (28.6%)**
**Maternal**								
Families/mothers	30	49	36	9	-	-	-	5
Single pregnancy only	17	28	20	4	-	-	-	1
Pregnancy MG undiagnosed	23/46 (50%)	36/89 (40.4%)	35/64 (54.7%)	1/21 (4.8%)	.004	11.484	2.337 – 64.986	0/6 (0%)
Pregnancy MG symptoms	20/41 (48.8%)	34/66 (51.5%)	22/52 (42.3%)	10/14 (71.4%)	.028	0.592	0.395 – 1.005	1/4 (25%)
Gestation(w): median; IQR (range)	38; 4 (27-40)	38; 5 (27-41)	36.4; 6 (27-41)	38; 4.3 (32-40)	-	-	-	38; 5 (27-41)
Prematurity (<37w)	10/37 (27%)	25/58 (43.1%)	20/40 (50%)	5/18 (27.8%)	0.134	1.800	0.889 – 4.137	2/4 (50%)
Antenatal death	7/46 (15.2%)	24/89 (27%)	23/64 (56.3%)	1/21 (4.8%)	0.018	7.547	1.495 – 43.026	1/6 16.7%
Antenatal contracture/AMC	12/46 (26.1%)	27/81 (33.3%)	24/61 (39.3%)	3/20 (15%)	0.062	2.623	1.014 – 7.711	1/5 (20%)
Polyhydramnios	22/43 (51.1%)	38/63 (60.3%)	27/45 (60%)	11/18 (61.1%)	0.935	0.982	0.669 – 1.612	1/4 (25%)
Reduced fetal movement	11/42 (26.2%)	24/68 (35.3%)	18/47 (38.3%)	6/19 (31.6%)	0.602	1.213	0.620 – 2.643	1/5 (20%)
IUGR	7/39 (17.9%)	10/51 (19.6%)	9/36 (25%)	1/15 (6.7%)	0.129	3.750	0.724 – 21.968	0/4 (0%)
Maternal Treatments								
AChEi	19/44 (43.2%)	37/78 (47.4%)	18/58 (31%)	17/18 (94.4%)	0.000	0.329	0.218 – 0.501	4/4 (100%)
Any IVIG/PLEX/Pred	15/46 (32.6%)	21/85 (24.7%)	-	6/21 (28.6%)	-	-	-	-
Corticosteroids	12/46 (26.1%)	15/78 (19.2%)	-	15/20 (75%)	-	-	-	-
IVIG	6/46 (13%)	7/79 (8.9%)	-	7/21 (33.3%)	-	-	-	-
PLEX	8/46 (17.4%)	12/79 (15.2%)	-	12/21 (57.1%)	-	-	-	-
Thymectomy[b]	14/46 (30.4%)	22/82 (26.8%)	9/61 (14.8%)	13/21 (61.9%)	0.000	0.0238	0.0123 – 0.479	3/6 (50%)
**Offspring**								
Male (n)	27/46	43/76	28/52	13/20	0.369	0.828	0.575 – 1.308	3/5
Death (inc. pre-natal)[c]	11/46 (23.9%)	41/89 (46.1%)	37/64 (57.8%)	4/21 (19%)	0.011	3.035	1.384 – 7.363	2/6 (33.3%)
Age follow up(y): median/IQR	7.5/14	2.33/11.8	1.5/11.86	5.25/14.89	-	-	-	5.5/11.83
**Neonatal-infancy**								
Hypotonia	35/36 (97.2%)	48/50 (96%)	29/30 (96.7%)	17/18 (94.4%)	0.819	1.024	0.876 – 1.304	4/4 (100%)
Facial weakness	36/37 (97.3%)	48/49 (98%)	31/31 (100%)	15/16 (93.8%)	0.524	1.067	0.938 – 1.395	4/4 (100%)
Craniofacial dysmorphism	24/31 (77.4%)	42/51 (82.4)	35/39 (89.7%)	7/12 (58.3%)	0.081	1.538	1.069 – 2.817	2/4 (50%)
Contractures/AMC[d]	31/46 (67.4%)	64/87 (73.6%)	56/64 (87.5%)	8/21 (38.1%)	0.002	2.297	1.452 – 4.229	3/6 (50%)
Respiratory support/intubation	27/37 (73%)	41/54 (75.9%)	29/35 (82.9%)	11/18 (61.1%)	0.139	1.356	0.967 – 2.167	4/4 (100%)
Neonatal intubation	13/37 (35.1%)	20/48 (41.7%)	16/31 (51.6%)	4/17 (23.5%)	0.077	2.194	0.977 – 5.581	1/4 (25%)
Neonatal NIPPV	9/37 (24.3%)	9/48 (18.8%)	-	-	-	-	-	2/4 (50%)
Pulmonary hypoplasia	10/42 (23.8%)	20/58 (34.5%)	18/39 (46.2%)	2/19 (10.5%)	0.019	4.385	1.358 – 16.046	0/5 (0%)
Diaphragmatic paresis	5/35 (14.3%)	5/43 (11.6%)	4/29 (14.8%)	1/14 (7.1%)	0.478	1.931	0.325 – 12.346	0/2 (0%)

Pleural effusion/chylothorax	4/38 (10.5%)	4/55 (7.3%)	4/39 (10.3%)	0[f]/16 (0%)	0.261	3.759	0.381 – 38.640	0/3 (0%)
Pneumothorax	2/36 (5.6%)	3/53 (5.6%)	3/37 (8.1%)	0[f]/16 (0%)	0.348	3.080	0.297 – 32.616	0/3 (0%)
NGT feeding	33/34 (97%)	38/39 (97.4%)	24/24 (100%)	14/15 (93.3%)	0.542	1.071	0.914 – 1.425	3/3 (100%
Pyloric stenosis	3/37 (8.1%)	3/46 (6.5%)	2/29 (6.9%)	1/17 (5.9%)	0.878	1.172	0.156 – 9.013	1/4 (25%)
Cryptorchidism (males only)	8/24 (33.3%)	13/38 (34.2%)	8/27 (29.6%)	5/11 (45.5%)	0.315	0.652	0.301 – 1.598	0/2 (0%)
Inguinal hernia	7/38 (21.2%)	7/58 (12%)	2/40 (5%)	5/18 (27%)	0.019	0.180	0.045 – 0.774	1/4 (25%)
**Later features**								
Facial weakness	25/34 (73.5%)	35/47 (74.5%)	22/29 (75.9%)	11/16 (68.8%)	0.633	1.103	0.778 – 1.745	3/5 (60%)
Muscle weakness	14/32 (43.8%)	22/43 (51.2%)	15/27 (55.6%)	5/14 (35.7%)	0.246	1.556	0.793 – 3.525	2/5 (40%)
Jaw contracture/malocclusion	6/32 (18.8%)	6/40 (15%)	5/27 (18.5%)	1/13 (7.7%)	0.329	2.407	0.434 – 14.785	0/5 (0%)
Extraocular involvement	5/22 (22.7%)	7/26 (26.9%)	6/16 (37.5%)	1/10 (10%)	0.123	3.750	0.759 – 21.861	0/2 (0%)
Scoliosis	7/44 (15.9%)	15/77 (19.5%)	13/58 (22.4%)	2/19 (10.5%)	0.245	2.129	0.628 – 8.049	0/5 (0%)
Feeding difficulty	16/36 (44.4%)	24/45 (53.3%)	16/28 (57.1%)	8/17 (47.1%)	0.517	1.214	0.708 – 2.288	2/4 (50%)
PEG requirement	8/31 (25.8%)	9/36 (25%)	7/21 (33.3%)	2/15 (13.3%)	0.167	2.500	0.714 – 9.574	1/3 (33%)
VPI >1y	18/24 (75%)	21/29 (72.4%)	10/12 (83.3%)	9/15 (60%)	0.213	1.389	0.841 – 2.366	2/4 (50%)
Speech difficulty	22/32 (68.8%)	25/38 (65.8%)	14/20 (70%)	9/16 (56.3%)	0.413	1.244	0.765 – 2.180	2/4 (50%)
Hearing impairment	12/32 (37.5%)	12/33 (36.4%)	9/22 (40.9%)	3/11 (27.3%)	0.431	1.500	0.592 – 4.465	0/2 (0%)
CNS involvement	7/40 (17.5%)	10/50 (20%)	10/31 (32.3%)	0[f]/15 (0%)	0.038	10.333	1.210 – 100.267	0/3 (0%)
Tracheostomy	3/35 (8.6%)	3/44 (6.8%)	3/26 (11.5%)	0[f]/16 (0%)	0.218	4.358	0.421 – 45.561	0/4 (0%)
OSA/Restrictive defect	9/28 (32.1%)	9/29 (31%)	6/19 (31.6%)	3/10 (30%)	0.925	1.053	0.386 – 3.287	2/4 (0%)
[e]Respiratory support (d): median (range)/IQR	45 (2-1825)/ IQR 85 (n=27)	47 (2-1825)/ IQR 89 (n=34)	53 (3-1825)/ IQR 111 (n=24)	13 (2-150)/ IQR 56 (n=10)	.05 (Z=-1.94)	-	-	18 (8-150) IQR 111 (n=4)

Percentages derive from the total number of cases where this feature was reliably established. [a]pIMT = pregnancy immunotherapy; Thymectomy[b] performed before the pregnancy; [c]All postnatal deaths occurred during early infancy; [d]Contractures/AMC includes a range of cases with contractures in more than one limb up to cases within the severe Fetal Akinesia Deformation Spectrum (FADS) presentations; [e]Excludes day1 deaths; IQR, interquartile range; IUGR, intrauterine growth restriction as commented on in publication or if birth weight below 10th centile; CNS = central nervous system; NGT = nasogastric tube; d = days; w = weeks; MG = myasthenia gravis; NIPPV = non-invasive positive pressure ventilation; OSA = obstructive sleep apnoea; PEG = percutaneous endoscopic gastrostomy; VPI = velopharyngeal incompetence; RR = risk ratios of fIMT (none versus any) with [f]continuity correction for cells with 0 applied by adding the value 0.5 to both numerator and denominator of both groups; CI = 95% confidence interval (Wilson's). Respiratory support duration compared by Mann-Whitney U test. $P<0.05$ considered statistically significant.
